# Smoking, Smoking Cessation, and the Risk of Type 2 Diabetes among Japanese Adults: Japan Epidemiology Collaboration on Occupational Health Study

**DOI:** 10.1371/journal.pone.0132166

**Published:** 2015-07-22

**Authors:** Shamima Akter, Hiroko Okazaki, Keisuke Kuwahara, Toshiaki Miyamoto, Taizo Murakami, Chii Shimizu, Makiko Shimizu, Kentaro Tomita, Satsue Nagahama, Masafumi Eguchi, Takeshi Kochi, Teppei Imai, Akiko Nishihara, Naoko Sasaki, Tohru Nakagawa, Shuichiro Yamamoto, Toru Honda, Akihiko Uehara, Makoto Yamamoto, Ai Hori, Nobuaki Sakamoto, Chiro Nishiura, Takafumi Totsuzaki, Noritada Kato, Kenji Fukasawa, Ngoc M. Pham, Kayo Kurotani, Akiko Nanri, Isamu Kabe, Tetsuya Mizoue, Tomofumi Sone, Seitaro Dohi, the Japan Epidemiology Collaboration on Occupational Health Study Group

**Affiliations:** 1 Department of Epidemiology and Prevention, Center for Clinical Sciences, National Center for Global Health and Medicine, Tokyo, Japan; 2 Mitsui Chemicals, Inc., Tokyo, Japan; 3 Teikyo University Graduate School of Public Health, Tokyo, Japan; 4 Nippon Steel & Sumitomo Metal Corporation Kimitsu Works, Chiba, Japan; 5 JFE Steel Corporation, Kanagawa, Japan; 6 Mitsubishi Plastics, Inc., Tokyo, Japan; 7 All Japan Labour Welfare Foundation, Tokyo, Japan; 8 Furukawa Electric Co., Ltd., Tokyo, Japan; 9 Azbil Corporation, Tokyo, Japan; 10 Mitsubishi Fuso Truck and Bus Corporation, Kanagawa, Japan; 11 Hitachi, Ltd., Ibaraki, Japan; 12 YAMAHA CORPORATION, Shizuoka, Japan; 13 Department of Safety and Health, Tokyo Gas Co., Ltd., Tokyo, Japan; 14 Genkiplaza Medical Center for Health Care, Tokyo, Japan; 15 Mizuho Bank, Ltd., Tokyo, Japan; 16 Fuji Electric Co., Ltd., Kanagawa, Japan; 17 Ricoh Co., Ltd., Kanagawa, Japan; 18 Department of Epidemiology, Faculty of Public Health, Thai Nguyen University of Medicine and Pharmacy, Thai Nguyen Province, Vietnam; 19 National Institute of Public Health, Saitama, Japan; Legacy, Schroeder Institute for Tobacco Research and Policy Studies, UNITED STATES

## Abstract

**Aims:**

To examine the association of smoking status, smoking intensity, and smoking cessation with the risk of type 2 diabetes (T2D) using a large database.

**Methods:**

The present study included 53,930 Japanese employees, aged 15 to 83 years, who received health check-up and did not have diabetes at baseline. Diabetes was defined as fasting plasma glucose ≥126 mg/dl, random plasma glucose ≥200 mg/dl, HbA1c ≥6.5% (≥48 mmol/mol), or receiving medication for diabetes. Cox proportional-hazards regression models were used to investigate the association between smoking and the risk of diabetes.

**Results:**

During 3.9 years of median follow-up, 2,441 (4.5%) individuals developed T2D. The multivariable-adjusted hazard ratios (95% CI) for diabetes were 1 (reference), 1.16 (1.04 to 1.30) and 1.34 (1.22 to 1.48) for never smokers, former smokers, and current smokers, respectively. Diabetes risk increased with increasing numbers of cigarette consumption among current smokers (P for trend <0.001). Although the relative risk of diabetes was greater among subjects with lower BMIs (< 23 kg/m^2^), attributable risk was greater in subjects with higher BMIs (≥ 23 kg/m^2^). Compared with individuals who had never smoked, former smokers who quit less than 5 years, 5 to 9 years, and 10 years or more exhibited hazards ratios for diabetes of 1.36 (1.14 to 1.62), 1.23 (1.01 to 1.51), and 1.02 (0.85 to 1.23), respectively.

**Conclusions:**

Results suggest that cigarette smoking is associated with an increased risk of T2D, which may decrease to the level of a never smoker after 10 years of smoking cessation.

## Introduction

Tobacco smoking is a leading global disease risk factor, with nearly 6 million premature deaths, 6.9% years of life lost, and 5.5% disability-adjusted life-years (DALYs) in 2010 [1]. The global prevalence of tobacco smoking among adults is 22% [2]. In Japan, the prevalence of smoking has been declining as nearly 20% of adults were current tobacco smokers in 2012 [2]. Diabetes is a major health problem worldwide. Its current prevalence is approximately 8%, and it is predicted to rise to 10% by the 2035 [3]. An extensive body of literature has consistently found that cigarette smoking is a risk factor for diabetes. In a systematic review with meta-analysis based on evidence from 25 cohort studies, Willi et al. found that active smoking was associated with a higher incidence of type 2 diabetes (T2D), followed by past smokers, compared to never smokers [4]. A dose-response relationship was also observed between diabetes and the number of cigarettes smoked [4].

Although epidemiological data have consistently supported the role of smoking in the pathogenesis of T2D, some issues remain to be addressed. For one, given that obesity is a strong risk factor for T2D and smoking influences weight, the effect of smoking on T2D risk may differ according to the level of obesity. However, the few studies that have addressed this issue reported inconsistent results [5–7]. Thus, it is unclear how obesity modifies the association between smoking and the risk of T2D. Another important issue is the effect of smoking cessation. As quitting smoking frequently accompanies weight gain and can worsen some diabetic symptoms [8], a concern has been raised as to whether smoking cessation increases the risk of T2D. Although some [9–13] but not all [6, 14] studies reported an increase in the risk of T2D during a few years after smoking cessation, most studies have consistently shown that the risk of diabetes decreased to the non-smoker level after a long-period of smoking cessation [9–15]. These findings, however, may be limited due to small sample size [6, 10], inclusion of men only [6, 9, 10], women only [12, 14], or self-reports of the diagnosis of diabetes [10, 12, 13, 15]. Herein, we examined the prospective association of smoking status, smoking intensity, and smoking cessation with the risk of T2D in a large cohort of Japanese workers.

## Methods

### Study design

The Japan Epidemiology Collaboration on Occupational Health (J-ECOH) Study is an on-going multi-center study among workers of 12 companies from various industries. Researchers collected several types of health-related data including general health examinations, which all employees are obliged to undergo each year under the health and safety law in Japan. As of August 2013, nine participating companies provided health check-up data for the period January 2008 to December 2012 or April 2008 to March 2013. The date of the earliest examination (mostly in 2008) was regarded as baseline, but if the 2008 dataset of a company contained a large number of missing data, the date of 2009 or 2010 examinations was considered as baseline for the company. Outcome of the present prospective analysis was determined using data of health check-ups after the baseline through 2013.

### Ethics statement

Detail of ethical procedure of J-ECOH study has been described elsewhere [16]. In short, the objective and procedure of the study was announced in each company by using posters. The requirement for written informed consent was waived. Participants did not provide their verbal or written informed consent to join the study but were allowed to refuse their participation. This procedure conforms to the Japanese Ethical Guidelines for Epidemiologic Research [17], where informed consent from each participants is not necessarily required for observational studies using existing data. The study protocol was approved by the Ethics Committee of the National Center for Global Health and Medicine, Japan.

### Study population

Of the 80,469 subjects (67,472 men and 12, 997 women aged 15 to 84 years) with baseline data, we excluded subjects in one company (n = 141) for which there was no information on former smoking. Of the remaining eight companies (n = 80,328), we excluded 14,132 subjects with no data on glucose measurement, 5,027 subjects with diabetes mellitus, 2,562 subjects with missing data on smoking, and 15 subjects with missing covariates that were used in the main analysis including body mass index (BMI), blood pressures, and drugs for hypertension. Some of the excluded subjects met two or more exclusion criteria. Of the remaining 58,592 subjects, we excluded 4,662 subjects who did not attend any subsequent health check-ups or attend but did not receive glucose measurements at the health check-up setting. Finally, 53,930 subjects remained for our first set of analyses to assess the association between the risk of diabetes among former and current smokers compared to that for never smokers. In the second set of analyses to assess the association between smoking intensity among current smokers (number of cigarettes smoked per day) and the risk of diabetes, data were available for six companies (n = 36,756). In the third set of analysis, which focused on duration of smoking cessation and the risk of diabetes, data were available for two major companies (n = 38,557).

### General health examination

Height and weight were measured using scale in a light cloth and without shoes. BMI was calculated as the body weight in kilograms divided by the square of body height in meters. Waist circumference at umbilical level was measured using tape in a standing position. Blood pressure was measured in a sitting position. Hypertension was defined as a systolic blood pressure (SBP) ≥140 mm Hg, a diastolic blood pressure (DBP) ≥90 mm Hg, or use of antihypertensive medication. History of disease and health-related lifestyle were ascertained via a questionnaire.

### Laboratory measurements

Plasma glucose was measured using an enzymatic method in seven companies and a glucose oxidase preoxidative electrode method in one company. Glycated hemoglobin (HbA1c) was measured by latex agglutination immunoassay in five companies, HPLC method in two companies, and enzymatic method in one company. HbA1c was measured according to a method of the Japan Diabetes Society and was converted to the National Glycohemoglobin Standardization Program (NGSP) equivalent value (%) using formula HbA1c (%) = 1.02 × HbA1c (Japan Diabetes Society) (%) + 0.25%.

### Smoking status

Smoking status (never, former, or current) was identified from the self-administered questionnaires at baseline. In six companies, the number of cigarettes smoked per day was also asked. In four companies, the total number of cigarettes smoked per day was reported as a continuous variable, while in the other two companies, the respondents selected from among three options (≤10, 11 to 20, or >20 cigarettes per day). Similarly, those who reported themselves as former smokers, a further question regarding number of years since quitting was asked. In one company, we calculated years since quitting before baseline as age at first visit minus the recalled age at quitting, and in another company, years since quitting was self-reported. From these data, we classified data on years of smoking cessation into three categories <5, 5 to 9, or ≥10 years.

### Diagnosis of type 2 diabetes and prediabetes

T2D and prediabetes were diagnosed according to the American Diabetes Association criteria [18]. Diabetes was defined as a fasting plasma glucose ≥126 mg/dl; random plasma glucose ≥200 mg/dl; HbA1c ≥ 6.5% (≥48 mmol/mol), or under medical treatment for diabetes. Individuals without diabetes at baseline who met any of these conditions in the subsequent health check-ups were considered to have incident T2D. Prediabetes was defined as a HbA1c of 5.7 to 6.4% (39 to 46 mmol/mol).

### Other covariates

In one major company, data on shift work, alcohol consumption, sleep duration, leisure-time physical activity, and family history of diabetes were also obtained via a self-administered questionnaire. These variables were adjusted for in a sensitive analysis.

### Statistical analyses

To assess the statistical significance of the differences in background characteristics of the study population across categories for the three smoking statuses, we used analysis of variance and Pearson chi-square test. Imputation, based on a regression model, was used to estimate missing values from known values to account for missing data [19] for waist circumference (missing in 5.2% of respondents). Age, sex, and BMI were included as covariates in the imputation.

Person-time was calculated from the date of the baseline examination to the date of the first diagnosis of diabetes in a subsequent examination or to the date of the last examination, whichever occurred first. We estimated age-adjusted incidence rate per 1,000 person-years and calculated the attributable risk for current and former smokers by subtracting age-adjusted incidence rate of never smokers from those of current or former smokers, respectively. We used Cox proportional hazards model to estimate hazard ratios (HRs) for incident diabetes by categories of smoking status, considering never smoker as the reference category. The first model was adjusted for baseline age (y, continuous), sex, and worksite. The second model was additionally adjusted for hypertension (yes or no), waist circumference (cm, continuous), and BMI (kg/m^2^, continuous). In one company (n = 32,373) where detailed information on lifestyle was available, we conducted a sensitivity analysis by adding to the second model alcohol intake (<23 or ≥23 g ethanol/d), shift work (yes or no), sleeping time (<6, 6 to <7, or ≥7 h/d), physical activity (<150 or ≥150 min/wk), and family history of diabetes (yes or no). We performed a stratified analysis according to BMI (<23 or ≥23 kg/m^2^). We repeated the above analysis after excluding 888 subjects with chronic diseases including stroke, angina pectoris, myocardial infarction, ischemic heart disease, and cancer. We also conducted several stratified analyses according to age (<45 and ≥45 years), prediabetes status, and hypertension status. Interaction terms between smoking status and the above-mentioned stratifying variables (dichotomous) were created and included in the model to assess statistical interactions. P for interaction was examined by using the likelihood ratio test.

Multivariable-adjusted HRs for incident diabetes by categories of smoking intensity who smoked 1 to 10, 11 to 20, and >20 cigarettes per day, compared with persons who never smoked, were estimated. Tests for trends were performed by assigning the categories of smoking intensity as ordinal numbers and modelling this variable as a continuous variable. To assess the effects of smoking cessation on the risk of diabetes, we stratified the study population into 5 smoking categories: never smokers, former smokers (years since quitting ≥10, 5 to 9, <5 years), and current smokers. All analyses were performed using Stata version 13.1 (StataCorp, College Station, Texas, USA).

## Results

Table 1 shows baseline characteristics of the study population by smoking status. Former smokers were older, had a higher BMI, larger waist circumference, hypertension, a family history of diabetes, and were physically more active than their counterparts. Current smokers were more likely to be shift workers and alcohol drinkers than never smokers and past smokers.

**Table 1:** Characteristics of study population without diabetes at baseline.

Characteristic	Never smoker	Former smoker	Current smoker	P value^1^
n	23189	10162	20579	
Men, %	72.3	96.3	95.5	<0.001
Age (years)	43.0 ± 10.2^2^	46.6 ± 9.1	43.2 ± 9.6	<0.001
BMI (kg/m^2^)	22.9 ± 3.0	24.0 ± 2.9	23.3 ± 3.3	<0.001
SBP (mmHg)	120.2 ± 14.8	122.8 ± 14.2	120.2 ± 14.4	<0.001
DBP (mmHg)	75.4 ± 10.7	78.2 ± 10.0	75.3 ± 10.2	<0.001
Hypertension, %	15.6	22.6	15.1	<0.001
Waist circumference (cm)	80.5 ± 9.2	83.5 ± 8.0	82.4 ± 8.8	<0.001
Shift work (yes, %)^3^	14.9	16.3	26.5	<0.001
Alcohol drinker (>23 g ethanol/d, %)^3^	11.1	28.5	50.9	<0.001
Sleeping time (<6 h/d, %)^3^	55.6	47.1	50.9	<0.001
Leisure-time physical activity (≥150 min/wk, %)^3^	13.2	17.3	12.7	<0.001
Family history of diabetes^3^, %	14.5	15.5	14.0	0.02

BMI, body mass index; SBP, systolic blood pressure; DBP, diastolic blood pressure.

^1^Calculated by using the Pearson chi-square test for categorical variables or analysis of variance F test for continuous variables at baseline.

^2^Mean ± SD (all such values).

^3^Data were available for 32373 subjects.

Table 2 shows adjusted HRs for incident diabetes according to baseline smoking status. During 3.9 years of median follow-up, 2,441 (4.5%) individuals developed T2D, corresponding to an incident rate of 12.8 per 1000 person-years. The age-adjusted incident rate was highest in current smokers (15.4), followed by past smokers (14.5) and never smokers (10.5). The attributable risk (absolute risk difference compared with never smokers) of T2D in current smokers and former smokers was 4.9 and 4.0 per 1000 person-years, respectively, and it was higher among subjects with a higher BMI than those with a lower BMI. After adjusting for age, sex, worksite, hypertension, BMI, and waist circumference, the HRs and 95% confidence interval (CI) for incidence of diabetes were 1.16 (1.04 to 1.30) in former smokers and 1.34 (1.22 to 1.48) in current smokers compared with never smokers. In one company, where detailed occupational and lifestyle data were available, a similar association was observed after further adjustment for alcohol intake, shift work, sleep time, physical activity, and family history of diabetes. The HRs (95% CI) were 1.00, 1.16 (1.02 to 1.33) and 1.31 (1.16 to 1.47) for never smoker, former smoker, and current smoker, respectively.

**Table 2:** Adjusted hazard ratios (95% CI) for incidence diabetes according to baseline smoking status.

	Never smoker	Former smoker	Current smoker
n	23189	10162	20579
Cases	799	568	1074
Incidence (/1000 person-years)	9.7	16.1	14.7
Age-adjusted incidence	10.5	14.5	15.4
Attributable risk^1^	-	4.0	4.9
Model 1^2^	1.00	1.20 (1.07–1.34)	1.31 (1.19–1.44)
Model 2^3^	1.00	1.16 (1.04–1.30)	1.34 (1.22–1.48)
Model 3^4^ ^,^ ^5^	1.00	1.16 (1.02–1.33)	1.31 (1.16–1.47)
****BMI <23 kg/m**^**2**^**			
n	12936	4570	10414
Cases	215	149	325
Incidence (/1000 person-years)	4.7	9.3	8.7
Age-adjusted incidence	5.3	8.2	9.0
Attributable risk^1^	-	2.9	3.7
Model 1^2^	1.00	1.33 (1.07–1.65)	1.50 (1.25–1.79)
Model 2^3^	1.00	1.27 (1.02–1.59)	1.49 (1.24–1.78)
Model 3^4^ ^,^ ^5^	1.00	1.22 (0.94–1.59)	1.37 (1.10–1.72)
****BMI ≥23 kg/m**^**2**^**			
n	10253	5592	10165
Cases	584	419	749
Incidence (/1000 person-years)	16.2	21.7	21.0
Age-adjusted incidence	16.9	20.1	22.3
Attributable risk^1^	-	3.2	5.4
Model 1^2^	1.00	1.12 (0.99–1.28)	1.28 (1.14–1.43)
Model 2^3^	1.00	1.11 (0.97–1.26)	1.26 (1.13–1.41)
Model 3^4^ ^,^ ^5^	1.00	1.13 (0.96–1.32)	1.25 (1.09–1.43)
P for interaction^6^		0.13	0.07

CI, confidence interval; BMI, body mass index.

^1^Difference in age-adjusted incidence rate per 1000 person-years between never smoker and current or former smoker.

^2^Adjusted for age (years), sex, and worksite.

^3^Adjusted for all factors in model 1 plus BMI (kg/m^2^), waist circumference (cm), and hypertension (yes or no).

^4^Adjusted for all factors in model 2 plus alcohol intake (<23 g ethanol/d or ≥ 23 g ethanol/d), shift work (yes or no), sleeping time (<6 h/d, 6–7 h/d, or ≥7 h/d), leisure-time physical activity (<150 min/wk or ≥150 min/wk), family history of diabetes (yes or no).

^5^Data were available for 32373 subjects.

^6^P for interaction for dichotomized variable of BMI (<23 or ≥23 kg/m^2^) on the association between smoking and type 2 diabetes was based on model 2 and was examined using the likelihood ratio test.

We performed a stratified analysis according to BMI (BMI<23 kg/m^2^ or BMI≥23 kg/m^2^) (Table 2). A stronger association was observed among individuals with lower BMIs (<23 kg/m^2^) than for those with higher BMIs (BMI≥23 kg/m^2^). Among subjects with lower BMIs, the HRs (95% CI) were 1.27 (1.02 to 1.59) for former smokers and 1.49 (1.24 to 1.78) for current smokers compared to never smokers. Among subjects with higher BMIs, the corresponding HRs (95% CI) were 1.11 (0.97 to 1.26) and 1.26 (1.13 to 1.41). In analyses excluding subjects with chronic diseases, a similar association was found among overall subjects and among each BMI sub-group.

In the stratified analysis according to age, a stronger association was observed among comparatively older individuals (≥45 years) than among those with younger individuals (<45 years) (S1 Table). Among older individuals, the HRs (95% CI) were 1.24 (1.09–1.41) for former smoker and 1.38 (1.22–1.55) for current smoker, as compared to never smoker. Among younger individuals the corresponding figures were 0.95 (0.76–1.20) and 1.23 (1.04–1.45). In stratified analyses according to prediabetes or hypertension status, the risk of diabetes among former and current smokers was significantly increased only among those without prediabetes or hypertension at baseline.

Table 3 shows adjusted HRs for incident diabetes according to baseline smoking intensity. The risk of diabetes increased significantly as the number of cigarettes smoked per day increased (P for trend <0.001). In a multivariable adjusted model, compared with persons who never smoked, the HRs of diabetes among persons who smoked 1 to 10, 11 to 20, and more than 20 cigarettes per day were 1.31 (1.10 to 1.58), 1.36 (1.22 to 1.52), and 1.50 (1.25 to 1.80), respectively. We also estimated HRs according to smoking intensity among those with lower and higher BMIs. The risk of diabetes increased significantly as the number of cigarettes smoked per day increased both among subjects with lower and higher BMIs (P for trend <0.001). However, the association was stronger among individuals with lower BMIs than among those with higher BMIs.

**Table 3:** Adjusted hazard ratios (95% CI) for incidence diabetes according to baseline smoking intensity^1^.

	**n**	**Cases**	**Incidence**	**Age-adjusted incidence**	**Attributable risk** ^**2**^	**Model 1** ^**3**^	**Model 2** ^**4**^
****Total****	36756						
Never smoker	19324	707	10.4	11.2	-	1.00	1.00
Current smoker (cigarettes smoked/d)							
1–10	3880	154	11.7	13.5	2.3	1.23 (1.03–1.46)	1.31 (1.10–1.58)
11–20	11604	660	15.7	16.9	5.7	1.37 (1.22–1.53)	1.36 (1.22–1.52)
21+	1948	149	21.5	20.8	9.6	1.51 (1.26–1.81)	1.50 (1.25–1.80)
P for trend						<0.001	<0.001
****BMI <23 kg/m**^**2**^**	23432	617					
Never smoker	10739	194	5.1	5.7	-	1.00	1.00
Current smoker (cigarettes smoked/d)							
1–10	2163	47	6.3	7.7	2.0	1.33 (0.96–1.84)	1.35 (0.98–1.87)
11–20	5807	204	9.6	10.5	4.8	1.50 (1.22–1.85)	1.49 (1.21–1.83)
21+	851	42	13.7	12.4	6.7	1.74 (1.24–2.46)	1.74(1.23–2.45)
P for trend						<0.001	<0.001
****BMI ≥23 kg/m**^**2**^**	21971	1566					
Never smoker	8585	513	17.0	17.8	-	1.00	1.00
Current smoker (cigarettes smoked/d)							
1–10	1717	107	18.9	20.9	3.1	1.26 (1.02–1.56)	1.28 (1.03–1.58)
11–20	5797	456	21.8	23.9	6.1	1.28 (1.12–1.45)	1.28(1.12–1.45)
21+	1097	107	27.7	28.4	10.6	1.49 (1.20–1.84)	1.39 (1.12–1.72)
P for trend						<0.001	<0.001
^5^P for interaction							0.07

CI, confidence interval; BMI, body mass index.

^1^Analyses excluded former smoker (n = 8647).

^2^Difference in age-adjusted incidence rate per 1000 person-years between never smoker and current smoker.

^3^Adjusted for age (years), sex, and worksite.

^4^Adjusted for all factors in model 1 plus BMI (kg/m^2^), waist circumference (cm), and hypertension (yes or no).

^5^P for interaction for dichotomized variable of BMI (<23 or ≥23 kg/m^2^) on the association between smoking and type 2 diabetes was based on model 2 and was examined using the likelihood ratio test

**Fig 1:**
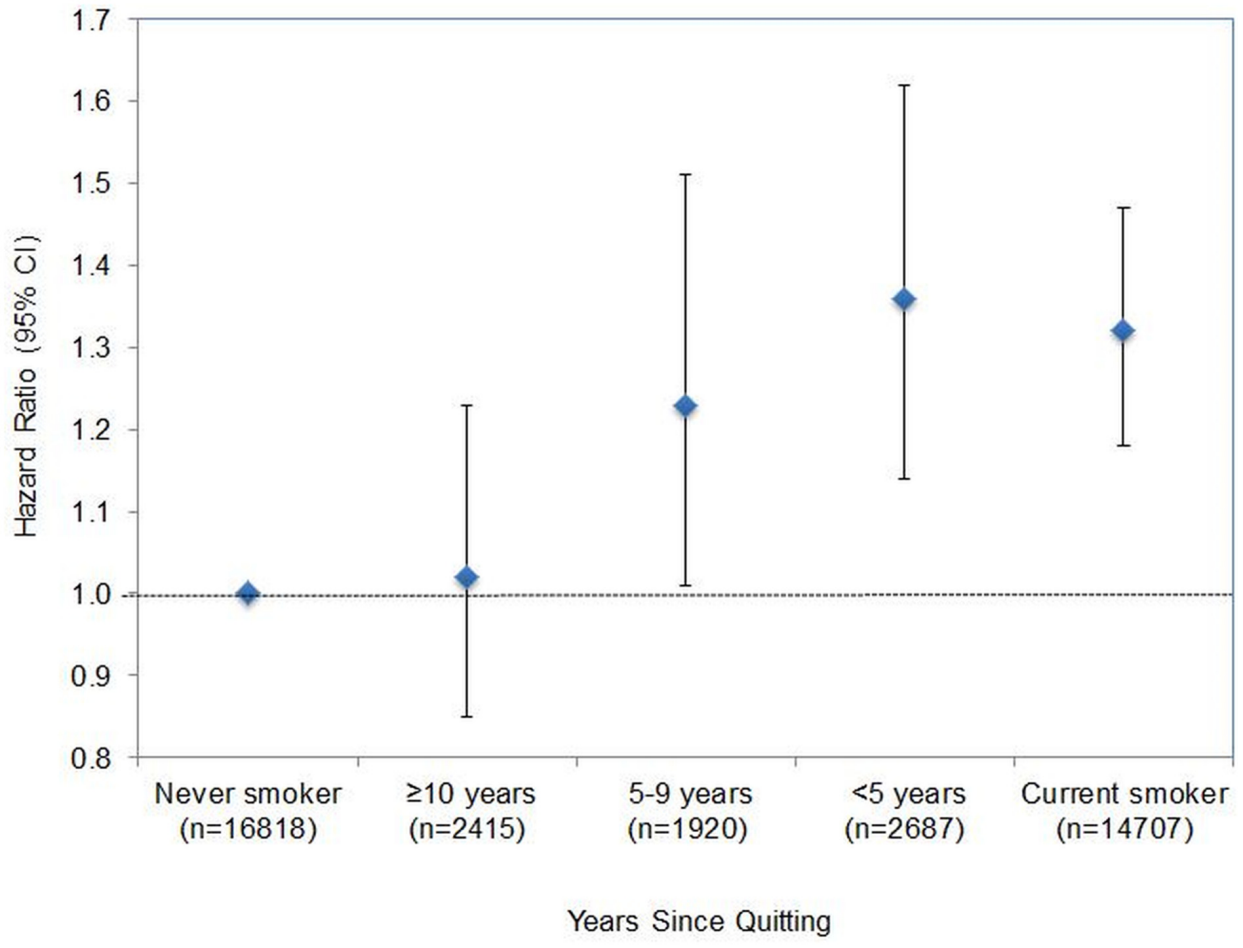
Adjusted hazard ratio for incident diabetes by years since quitting before baseline. Estimates are adjusted for age, sex, worksite, and hypertension. Bars indicate 95% confidence intervals. Never smokers are the reference group.

Fig 1 shows HRs for incident diabetes by years since quitting before baseline. In a multivariable model adjusted for age, sex, worksite, and hypertension, HRs (95% CI) for diabetes were 1.00 (reference), 1.36 (1.14 to 1.62), 1.23 (1.01 to 1.51), and 1.02 (0.85 to 1.23) for never smokers, former smokers who quit less than 5 years before, 5 to 9 years before, and 10 years or more before the baseline visit, respectively. Although we observed a slightly higher BMI among those who had quit smoking (baseline mean BMI: 23.53, 23.56, and 23.52 kg/m^2^ for those who quit <5, 5 to 9, and ≥10 years, respectively) than among current smokers (baseline mean BMI: 23.23 kg/m^2^), the association was materially unchanged after additional adjustments for BMI: the corresponding HRs (95% CI) were 1.00 (reference), 1.35 (1.13 to 1.60), 1.18 (0.97 to 1.45), and 1.01 (0.84 to 1.21).

## Discussion

Using a large database and an objective measurement of blood glucose, we have demonstrated that cigarette smoking is associated with an increased risk of T2D. Among current smokers, the risk of diabetes increased significantly as the number of cigarettes smoked per day increased. Participants who had quit smoking less than 5 years before baseline had a similar risk of diabetes than those who continued smoking, but the risk dissipated steadily as the time since smoking cessation increased.

Our findings of an elevated risk of diabetes among current smokers are consistent with the findings of a meta-analysis of 25 prospective cohort studies on active smoking and T2D [4] and findings from the cohort studies published after the systematic review [11, 12, 14, 20]. The significant positive association between the number of cigarettes smoked per day and the risk of diabetes in our study is consistent with the findings of a number of recent studies [12, 20, 21]. The magnitude of the increased risk is also comparable to the estimate from the meta-analysis. We found a stronger association with respect to the relative risk between current smoking and the risk of T2D among subjects with lower BMIs (BMI<23 kg/m^2^) than those with higher BMIs (BMI≥23 kg/m^2^). Previously, studies among middle-aged Japanese [5] and US [7] men reported a stronger association among normal-weight participants than over-weight or obese participants, whereas another Japanese study reported the opposite data [22]. In spite of the inconsistencies among studies for the relative risk estimate, all previous studies, as well as the current study, have consistently shown that the risk attributable to smoking or the risk difference between non-smoker and smoker was greater among over-weight persons than among normal-weight persons. Given a greater absolute impact of smoking on T2D risk among over-weight persons, the avoidance of smoking together with weight control would be advised for obese smokers.

In the present study, the risk of diabetes among former smokers was higher than it was for never smokers, a finding consistent with those of the meta-analysis [4] and with recently published studies [11, 12, 20]. However, results for diabetic risk for recent quitters relative to current smokers vary among studies. The degree of increase of diabetic risk was similar between recent quitters (quit <5 years before baseline) and current smokers in the present study. Some studies in Japan [15], Korea [9], and US [11, 12], however, have reported a sizable increase (>20%) in the risk of diabetes risk among recent quitters (quit <5 years) compared with current smokers. In contrast, other studies in Japan [6] and US [14] have reported a lower risk of diabetes among recent quitters than among current smokers. Such inconsistencies may be ascribed to methodological differences among studies, including the definition of recent quitter (some studies defined former smokers based on the information of smoking status at baseline only, while others also used information during follow-up) and the length of the follow-up period (some studies followed for ≤5 years, while others followed for >5 years).

The aging of population is a significant driver of diabetes epidemic, but few data are available that compared the impact of smoking on diabetes risk between different age groups. In the present study, a significantly increased risk of diabetes associated with current smoking was observed among both older (≥45 years) and younger (<45 years) subjects, whereas the risk associated with former smoking was statistically significantly increased only among older subjects. The duration of smoking may partly responsible for the differential association for former smoking. We found that the mean duration of smoking was much longer among older former smokers (20.7 years) than that among younger former smokers (12.0 years). Previously, in a study among middle aged Japanese, an increased risk of diabetes was observed among quitters who had smoked cigarettes for more than 30 to 40 years, but not among those who had smoked less than 30 years [15].

Prediabetes is considered as a high risk state of developing diabetes, and hypertension is considered as an important risk factor for diabetes. In our previous cross-sectional study, the prevalence of diabetes was about two times higher among subjects with hypertension than those without [23]. In the present study, participants with prediabetes or hypertension had much higher incidence rate of diabetes than those without these conditions. Besides, we found a weaker association, in terms of relative risk, between current smoking and diabetes among subjects with prediabetes or hypertension than among those without each of these conditions. Similarly, a previous US study reported a weaker association between current smoking and diabetes risk among subjects with impaired glucose tolerance than among those with normal glucose tolerance [24]. More important finding of the present study in terms of prevention was that subjects with prediabetes or hypertension showed somewhat greater absolute risk associated with smoking than did subjects without these conditions. Therefore, avoidance of smoking should be advocated irrespective of glycemic or blood pressure status.

In the present study, the risk of diabetes among those who had quit smoking decreased as the number years since smoking cessation increased up to 10 years. After 10 years or more of cessation, the risk levels was the same as that of never smokers. Similarly, most [9–15] but not all [6] studies found a gradual reduction in diabetic risk over time after smoking cessation. Moreover, the majority of previous studies on this issue reported diabetic risk among quitters with 10-years or more of cessation was comparable to that of never smokers [11, 13–15]. Taken together, the increased risk of T2D associated with smoking may gradually decrease after smoking cessation and totally disappear after at least 10 years of smoking cessation.

The underlying mechanism by which smoking increases the risk of T2D is unclear, although several potential mechanisms have been suggested. First, active smoking has been related to various systemic effects including oxidative stress, systematic inflammation, and endothelial dysfunction [25, 26], all of which have been linked to insulin resistance [27, 28]. Second, nicotine in cigarettes has a direct toxic effect on beta cell function [29]. Third, although smoking tends to decrease weight, it leads to central adiposity [30, 31], which has been linked to inflammation [32] and insulin resistance [33].

The strengths of our study include its prospective design, large sample size, and diagnoses of diabetes using both fasting glucose and HbA1c. Despite these strengths, our study has several limitations that deserve mention. First, the follow-up period of our study was relatively short. The present findings in terms of incidence rate and relative risk, however, are comparable to those of a meta-analysis [4] and a long-term prospective study in Japan [34]. Second, no information was collected on diet or weight change due to smoking cessation, and hence, we were unable to control for or assess the joint effect of these factors on the risk of T2D. Third, the method of blood glucose and HbA1c measurement differed among companies. Given the highest level of quality control achieved in all of the participating companies, however, measurement bias is unlikely. Fourth, although we adjusted for putative and potential confounders, the possibility of residual confounding and unmeasured factors cannot be ruled out. Finally, our study subjects were employees from large companies, and thus the findings might not be generalizable to populations with different backgrounds or to non-working populations.

In conclusion, the present study found an increased risk of T2D in both current and former smokers, with a clear dose-response relationship between smoking intensity and diabetic risk in current smokers. Although recent quitters did not have a lower risk of T2D than current smokers, long-term quitters (10 or more years of cessation) had a risk that was comparable to that of never smokers. These findings greatly strengthen the evidence regarding the relationship between smoking and T2D, and they provide a rationale for the urgent implementation of tobacco control programs, especially in countries such as Japan where tobacco smoking is still prevalent.

## Supporting Information

S1 Table Adjusted hazard ratios (95% CI) for incidence diabetes according to baseline smoking status stratified according to age, prediabetes, and hypertension. (DOCX)
