# Correction: Smoking, Smoking Cessation, and the Risk of Type 2 Diabetes among Japanese Adults: Japan Epidemiology Collaboration on Occupational Health Study

**DOI:** 10.1371/journal.pone.0137039

**Published:** 2015-08-25

**Authors:** Shamima Akter, Hiroko Okazaki, Keisuke Kuwahara, Toshiaki Miyamoto, Taizo Murakami, Chii Shimizu, Makiko Shimizu, Kentaro Tomita, Satsue Nagahama, Masafumi Eguchi, Takeshi Kochi, Teppei Imai, Akiko Nishihara, Naoko Sasaki, Tohru Nakagawa, Shuichiro Yamamoto, Toru Honda, Akihiko Uehara, Makoto Yamamoto, Ai Hori, Nobuaki Sakamoto, Chihiro Nishiura, Takafumi Totsuzaki, Noritada Kato, Kenji Fukasawa, Ngoc M. Pham, Kayo Kurotani, Akiko Nanri, Isamu Kabe, Tetsuya Mizoue, Tomofumi Sone, Seitaro Dohi, Japan Epidemiology Collaboration on Occupational Health Study Group

The twenty-second author’s name is spelled incorrectly. The correct name is: Chihiro Nishiura.
