# Supplementary material for: Smoking, Smoking Cessation, and the Risk of Type 2 Diabetes among Japanese Adults: Japan Epidemiology Collaboration on Occupational Health Study
Source: PLoS One. 2015 Jul 22;10(7):e0132166. doi: 10.1371/journal.pone.0132166 (PMC4511672; doi:10.1371/journal.pone.0132166)
Supplement: S1 Table — (DOCX) [file pone.0132166.s001.docx]

**S1 Table** Adjusted hazard ratios (95% CI) for incidence diabetes according to baseline smoking status stratified according to age, prediabetes, and hypertension.

|  | Never smoker | Former smoker | Current smoker |
| --- | --- | --- | --- |
| Age |  |  |  |
| Age ≥45 years |  |  |  |
| n | 10071 | 5978 | 9170 |
| Cases | 534 | 459 | 714 |
| Incidence (/1000 person-years) | 15.9 | 23.1 | 23.2 |
| Attributable risk^1^ | − | 7.2 | 7.3 |
| Model 1^2^ | 1.00 | 1.27 (1.12−1.45) | 1.32 (1.17−1.48) |
| Model 2^3^ | 1.00 | 1.24 (1.09−1.41) | 1.38 (1.22−1.55) |
| Age <45 years |  |  |  |
| n | 13118 | 4184 | 11409 |
| Cases | 265 | 109 | 360 |
| Incidence (/1000 person-years) | 5.5 | 7.1 | 8.5 |
| Attributable risk^1^ | − | 1.6 | 3.0 |
| Model 1^2^ | 1.00 | 0.98 (0.78−1.23) | 1.26 (1.07−1.48) |
| Model 2^3^ | 1.00 | 0.95 (0.76−1.20) | 1.23 (1.04−1.45) |
| P for interaction^4^ |  | 0.07 | 0.30 |
| Prediabetes status |  |  |  |
| Prediabetes^5^ |  |  |  |
| n | 4153 | 2223 | 4788 |
| Cases | 372 | 272 | 491 |
| Age-adjusted incidence | 25.1 | 33.6 | 30.1 |
| Attributable risk^1^ | − | 8.5 | 5.0 |
| Model 1^2^ | 1.00 | 1.17 (0.99−1.38) | 1.03 (0.90−1.19) |
| Model 2^3^ | 1.00 | 1.13 (0.96−1.32) | 1.06 (0.92−1.22) |
| Non-prediabetes |  |  |  |
| n | 19036 | 7939 | 15791 |
| Cases | 427 | 296 | 583 |
| Age-adjusted incidence | 7.0 | 9.6 | 11.0 |
| Attributable risk^1^ | − | 2.6 | 4.0 |
| Model 1^2^ | 1.00 | 1.19 (1.02−1.39) | 1.43 (1.26−1.63) |
| Model 2^3^ | 1.00 | 1.17 (1.01−1.37) | 1.49 (1.31−1.70) |
| P for interaction^4^ |  | 0.31 | 0.002 |
| Hypertension status  Hypertension^6^ |  |  |  |
| n | 3735 | 2359 | 3226 |
| Cases | 302 | 228 | 317 |
| Age-adjusted incidence | 24.9 | 28.8 | 30.3 |
| Attributable risk^1^ | − | 7.2 | 7.3 |
| Model 1^2^ | 1.00 | 1.10 (0.92−1.32) | 1.15 (0.98−1.35) |
| Model 2^3^ | 1.00 | 1.11 (0.93−1.33) | 1.16 (0.99−1.37) |
| Non-hypertension |  |  |  |
| n | 19454 | 7803 | 17353 |
| Cases | 497 | 340 | 757 |
| Age-adjusted incidence | 7.7 | 11.0 | 12.6 |
| Attributable risk^1^ | − | 3.3 | 5.4 |
| Model 1^2^ | 1.00 | 1.24 (1.07−1.43) | 1.46 (1.29−1.64) |
| Model 2^3^ | 1.00 | 1.18 (1.02−1.36) | 1.44 (1.28−1.62) |
| P for interaction^4^ |  | 0.26 | 0.03 |

CI, confidence interval; BMI, body mass index.

^1^Difference in incidence rate per 1000 person-years between never smoker and current or former smoker.

^2^Adjusted for age (years), sex, and worksite.

^3^Adjusted for all factors in model 1 plus BMI (kg/m^2^), waist circumference (cm), and hypertension (yes or no).

^4^P for interaction for dichotomized variables of age, prediabetes, and hypertension on the association between former and current smoker and diabetes was based on model 2 and was examined using likelihood ratio test.

^5^Prediabetes was defined as a HbA1c of 5.7 to 6.4% (39 to 46 mmol/mol).

^6^Hypertension was defined as a systolic blood pressure ≥140 mm Hg, a diastolic blood pressure ≥90 mm Hg, or use of antihypertensive medication.
